# A fully-virulent retargeted oncolytic HSV armed with IL-12 elicits local immunity and vaccine therapy towards distant tumors

**DOI:** 10.1371/journal.ppat.1007209

**Published:** 2018-08-06

**Authors:** Valerio Leoni, Andrea Vannini, Valentina Gatta, Julie Rambaldi, Mara Sanapo, Catia Barboni, Anna Zaghini, Patrizia Nanni, Pier-Luigi Lollini, Costanza Casiraghi, Gabriella Campadelli-Fiume

**Affiliations:** 1 Department of Experimental, Diagnostic and Specialty Medicine, University of Bologna, Bologna, Italy; 2 Department of Veterinary Medical Sciences, University of Bologna, Bologna, Italy; Louisiana State University Health Sciences Center, UNITED STATES

## Abstract

Oncolytic herpes simplex viruses (oHSVs) showed efficacy in clinical trials and practice. Most of them gain cancer-specificity from deletions/mutations in genes that counteract the host response, and grow selectively in cancer cells defective in anti-viral response. Because of the deletions/mutations, they are frequently attenuated or over-attenuated. We developed next-generation oHSVs, which carry no deletion/mutation, gain cancer-specificity from specific retargeting to tumor cell receptors—e.g. HER2 (human epidermal growth factor receptor 2)—hence are fully-virulent in the targeted cancer cells. The type of immunotherapy they elicit was not predictable, since non-attenuated HSVs induce and then dampen the innate response, whereas deleted/attenuated viruses fail to contrast it, and since the retargeted oHSVs replicate efficiently in tumor cells, but spare other cells in the tumor. We report on the first efficacy study of HER2-retargeted, fully-virulent oHSVs in immunocompetent mice. Their safety profile was very high. Both the unarmed R-LM113 and the IL-12-armed R-115 inhibited the growth of the primary HER2-Lewis lung carcinoma-1 (HER2-LLC1) tumor, R-115 being constantly more efficacious. All the mice that did not die because of the primary treated tumors, were protected from the growth of contralateral untreated tumors. The long-term survivors were protected from a second contralateral tumor, providing additional evidence for an abscopal immunotherapeutic effect. Analysis of the local response highlighted that particularly R-115 unleashed the immunosuppressive tumor microenvironment, i.e. induced immunomodulatory cytokines, including IFNγ, T-bet which promoted Th1 polarization. Some of the tumor infiltrating cells, e.g. CD4+, CD335+ cells were increased in the tumors of all responders mice, irrespective of which virus was employed, whereas CD8+, Foxp3+, CD141+ were increased and CD11b+ cells were decreased preferentially in R-115-treated mice. The durable response included a breakage of tolerance towards both HER2 and the wt tumor cells, and underscored a systemic immunotherapeutic vaccine response.

## Author summary

There is increasing interest in oncolytic viruses (OVs), following the approval of Oncovex^GM-CSF^, and the success of a number of them in clinical trials. Most OVs, particularly the oHSVs, are attenuated to varying degree. In contrast, the tropism-retargeted oHSVs are fully-virulent, highly effective oncolytic agents, and appear to be highly safe in mice. Up to now, it was unknown how efficacious the retargeted oHSVs are as immunotherapeutic agents. Here, the demonstration that they elicit local immune response and systemic therapy vaccine effects opens the possibility that the fully-virulent retargeted oHSVs may be highly efficacious oncolytic-immunotherapeutic agents.

## Introduction

Oncolytic viruses (OVs) meet the need for novel anticancer agents characterized by low toxicity and low negative impact on the quality of life of patients [1–4]. Oncolytic herpes simplex viruses (oHSVs) stand for their efficacy in a number of clinical applications [5,6]. The most successful oHSV, Oncovex^GM-CSF^, was approved against metastatic melanoma [7,8]. The Clinical trials.gov website lists 22 open or recently completed trials with oHSVs [9–13]. Much of the current interest in OVs stems from their immunotherapeutic properties. Thus, oHSVs, and OVs in general, boost the immune response to the tumor, exert a therapeutic vaccine effect with no requirement for the identification of the tumor-specific or patient-specific neoantigens [14–17]. In combination with checkpoint inhibitors (CPIs), they enhance the efficacy of the blockade therapy [18–21]. They can be engineered to express anti-checkpoint antibodies [22].

The oHSVs in clinical practice or trials are attenuated to varying degrees, and gain their cancer specificity from the attenuation [3,5,23,24]. In essence, safety was achieved at the expense of virulence. The attenuated oHSVs infect preferentially, but not exclusively, the cancer cells. Attenuation was attained through genetic engineering, as in the Δγ_1_34.5 viruses, including Oncovex^GM-CSF^, or through natural mutations [23,25,26]. In some examples, multiple deletions resulted in high attenuation, to the point that oHSVs replicated to limited extent even in the tumor cells [27] and were scarcely efficacious as single agents. In Oncovex^GM-CSF^, the attenuation is reversed by the immediate early expression of US11, a viral protein which counteracts protein kinase R [28]. To circumvent the attenuation effects, OVs are employed as vectors for the transgenic expression of cytokines or CPIs. Indeed, the first cytokine-expressing oHSV was designed by Martuza and Rabkin some 20 years ago [29]. Oncovex^GM-CSF^ is armed with GM-CSF, which activates APCs, boosts the immune response to the tumor, and enables a distant effect [23]. A key modulator of the cancer immune response is IL-12. This cytokine targets a variety of immune cells, activates effector cells, induces IFNγ secretion which boosts and sustains the immune response [30–32]. In humans, the systemic administration of IL-12 was marred by toxicity. The expression of IL-12 from OVs, in particular oHSVs, raised the hopes to benefit from local administration, without the toll of systemic toxicity. The IL-12-armed Δγ_1_34.5 oHSVs showed efficacy in preclinical models [19,29,33–37], and one of them is in clinical trial against glioblastoma multiforme [38].

An alternative approach to safety-through-attenuation centres on specific tropism for the cancer cells, achieved by retargeting the virus tropism to cancer-specific receptors of choice, and detargeting from the natural receptors [39–41]. The retargeted oHSVs carry no deletion/attenuation. In their target cancer cells they are fully virulent. Because they infect no other cell than the specifically-targeted cancer cells, they promise to be highly safe. When injected intraperitoneally, they caused no harm to tumor-free mice up to the maximum tested dosage (10^8^ PFU) [42]. The tumor cell receptor selected in our laboratory was HER2 (human epidermal growth factor receptor 2) [43–48] overexpressed in a number of cancers [49]. The HER2-retargeted oHSVs named R-LM249 and R-LM113 exerted a strong therapeutic efficacy in immunodeficient mice [42,50–52]. A single virus administration practically ablated tumor growth [42]. When administered intraperitoneally (i.p.) in a model of peritoneal carcinomatosis they rendered 60% of mice tumor-free [51]. The immunodeficient mouse model underscores the therapeutic effect against primary tumors, accounts for the oncolytic effect of the virus, but is inadequate to evaluate the immunotherapeutic effects. The central question that prompted this study was to what extent a fully virulent HER2-retargeted oHSV, armed with IL-12, exemplified here by R-115, was able to elicit a local immune response, lymphocytes migration to the tumor and activation, and ultimately local and distant immunotherapeutic efficacy. The question stemmed from intrinsic differences between retargeted oHSVs and the deleted/mutated oHSVs in clinical use. A major difference relates to the innate response, a phenomenon that also impacts on adaptive immunity. As mentioned, most oHSVs are defective in the synthesis of the γ_1_34.5 product, a protein that contrasts the host innate response to the virus [26]. Hence, these viruses are defective in counteracting the innate response. In contrast, fully virulent non-attenuated HSVs first elicit an innate response (e.g. secretion of IFN type I, TNF, etc.), but then dampen it through a number of molecular mechanisms (e.g. secretion of IL-10, IL-6) that limit the hostile microenvironment and ultimately favour viral replication [53–57]. It was thus unclear to what extent the regulation of the innate response put in place by a virulent oHSV would affect its ability to modify the immunosuppressive tumor microenvironment, and to elicit a strong adaptive durable response. Additional differences include the efficient replication and the lack of off-target infection. Specifically, the retargeted oHSVs replicate to near wt-virus yields in human target cancer cells [48], and fail to infect cells other than the targeted cancer cells [44]. In contrast, the currently employed oHSVs infect various cell types in the tumor bed. We report that R-LM113, and its IL-12 encoding derivative R-115 inhibited the growth of the primary treated tumor, completely prevented the growth of distant untreated tumors, elicited local and systemic immune response and thus induced a vaccine-like response. In all assays the IL-12-armed R-115 was more effective than the unarmed R-LM113.

## Results

### Choice of a murine cancer cell line, transgenically expressing HER2, permissive to HSV infection

**Fig 1:**
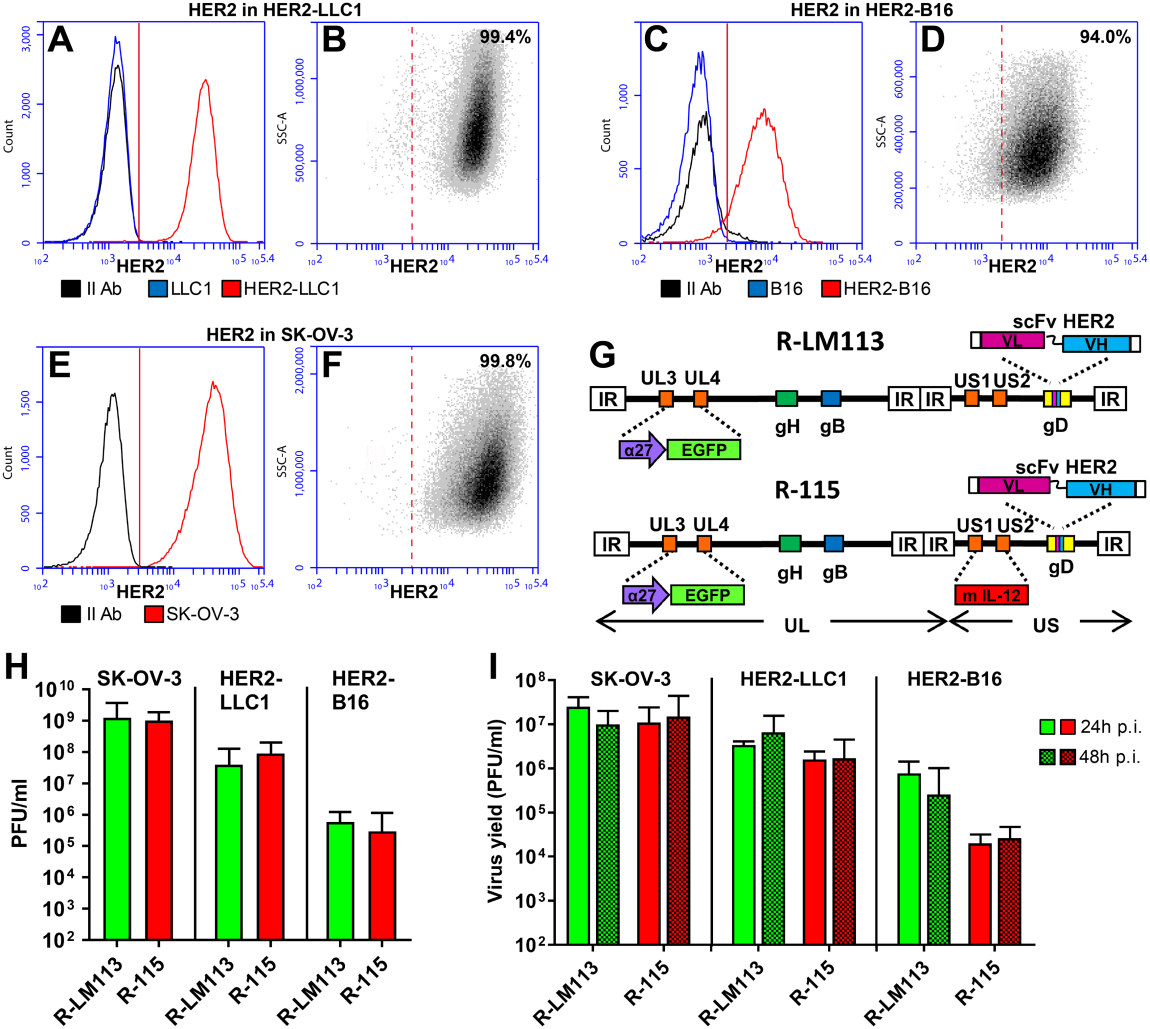
Properties of HER2-expressing murine tumor cells. (A—F) HER2 expression in HER2-LLC1 (A, B), HER2-B16 (C, D), and SK-OV-3 (E, F) cells, and in the wt LLC1 (A) and B16 (C) cells as controls. HER2 expression was detected by flow cytometry by means of anti-HER2 Ab. (A, C, E) X-axis, fluorescence intensity; y-axis, counts. HER2-LLC1 and HER2-B16, red; LLC1 and B16 wt, blue; the secondary anti-mouse Ab alone, black. The average fluorescence intensities of three independent determinations ± SD were 30420 ± 1155, 8589 ± 334, 43810 ± 1796 for HER2-LLC1, HER2-B16, and SK-OV-3 cells, respectively. (B, D, F) Homogeneity of the HER2-LLC1, HER2-B16 clonal cells, and of SK-OV-3 cells. X-axis, fluorescence intensity; y-axis, side scatter (SSC-A). Figures denote the percentage of cells positive to anti-HER2 Ab. (G) Schematic backbones of R-LM113 and R-115 genomes [44,60]. Both R-LM113 and R-115 carry the insertion of BAC (bacterial artificial chromosome) sequences and EGFP (enhanced green fluorescence) gene in the UL3-UL4 intergenic region, the deletion of the aa 6–38 region in gD for detargeting purposes and its replacement with the scFv (single chain antibody) to HER2 for retargeting purposes. In addition, R-115 carries the mIL-12 gene under the hCMV (human cytomegalovirus) promoter in the US1-US2 intergenic region. (H) Relative plating efficiency of R-LM113 and R-115 in SK-OV-3, HER2-LLC1 and HER2-B16 cells, measured as efficiency of plaque formation. Replicate aliquots of R-115 or R-LM113 were plated onto HER2-LLC1, HER2-B16 and SK-OV-3 cell monolayers, in triplicates. Plaques were scored three days later. (I) Yields of R-LM113 and R-115 in SK-OV-3, HER2-LLC1 and HER2-B16 cells. For each cell line replicate cultures were infected with the indicated viruses at 0.1 PFU/cell (as titrated in the respective cell line). Progeny virus was titrated in SK-OV-3 cells. In panels H and I, each column represents the average of triplicates ± SD.

A major difficulty encountered when switching from immunodeficient to immunocompetent mice is that murine cells are scarcely permissive to HSV infections, and the viral replication may be 2–3, or more, logs lower in murine cancer cells than in human cancer cells [58,59]. This is an obvious obstacle in the preclinical studies, and strongly underestimates the efficacy of oHSVs. Further limitations in our experimental model were that HER2-retargeted oHSV only infected HER2-expressing cells, and that the appropriate host for these cells are HER2-transgenic/tolerant mice. Here, we made use of the C57BL/6 HER2-transgenic/tolerant mice, although the parental strain is among the least sensitive to HSV. The murine B16 melanoma cells and the Lewis lung carcinoma (LLC1) cells were made HER2-transgenic by lentiviral transduction, selection with puromycin and single cell cloning. The HER2-LLC1 cells expressed HER2 at higher level than the HER2-B16 cells (Fig 1, compare A to C) and at similar level as the SK-OV-3 cells (Fig 1E), a HER2-expressing human ovary cancer cell line. The HER2-LLC1 and HER2-B16 cells were homogeneous clones (Fig 1B and 1D). SK-OV-3 are shown for comparison (Fig 1F). In both the HER2-LLC1 and HER2-B16 cells, the HER2 expression was stable for more than 30 consecutive passages.

HER2-LLC1 and HER2-B16 cells were compared for ability to support the replication of R-LM113 and R-115 [44,60]. The latter is a R-LM113 derivative, which expresses the murine interleukin 12 (mIL-12) (see Fig 1G for a schematic representation of R-LM113 and R-115 genomes), in the amount of 200–400 pg/10^5^ SK-OV-3 cells [60]. Fig 1H reports the plating efficiency of both viruses and shows that, on average, the amounts of viruses required to infect a single SK-OV-3, HER2-LLC1 and HER2-B16 cell were 1, 20, and 2500 PFUs, respectively. Based on these figures, we next carried out a virus growth experiment in the three cell lines. The cells were infected at 0.1 PFU/cell, according to the virus titre determined in the respective cell line. Under these conditions, R-LM113 and R-115 grew in HER2-LLC1 cells at higher yields than in HER2-B16 cells, and at one order of magnitude lower yields than in the susceptible human SK-OV-3 cells (Fig 1I).

### The HER2 transgenic/tolerant mouse model and safety of the HER2-retargeted R-LM113 and R-115

**Fig 2:**
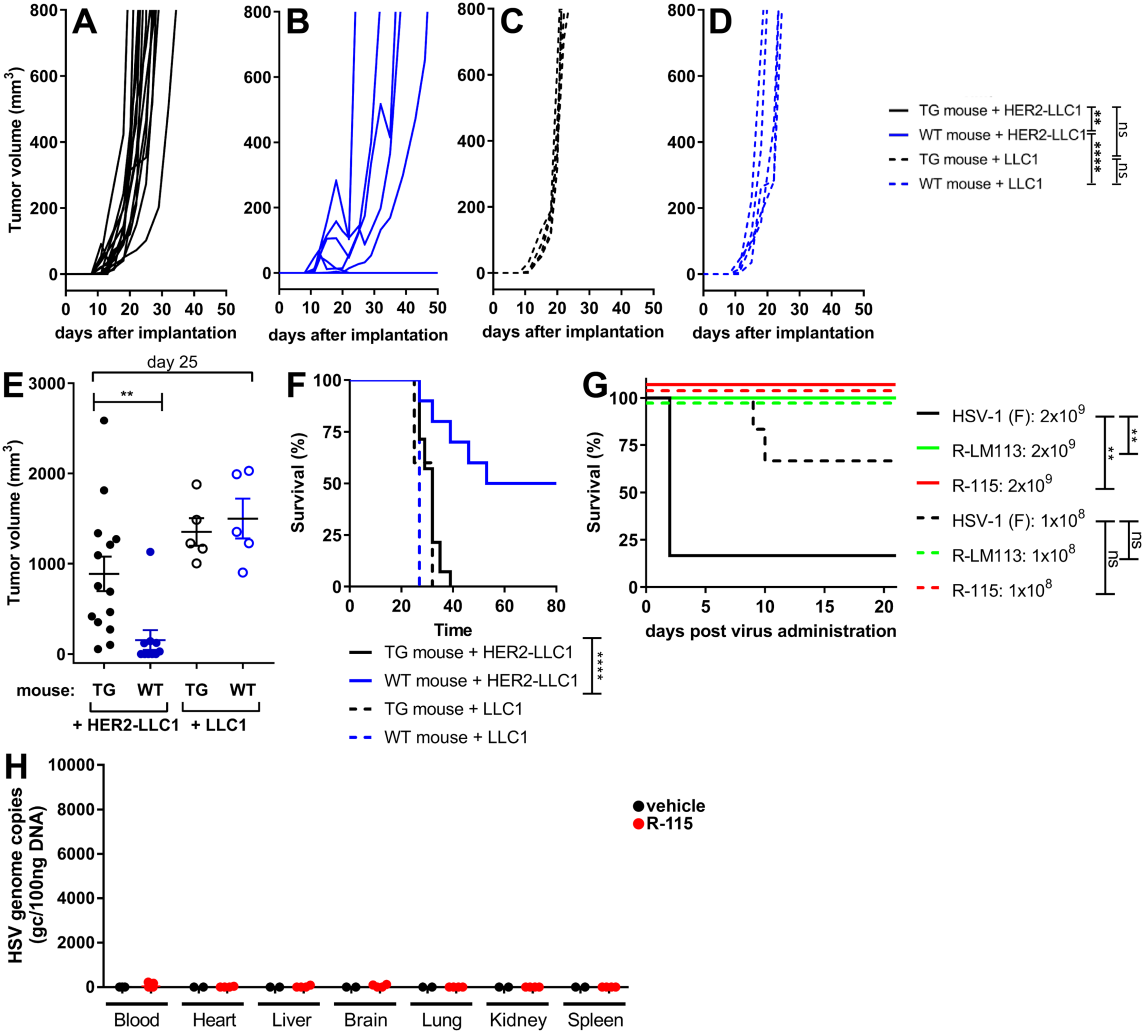
The HER2-transgenic/tolerant mouse model and safety of R-LM113 and R-115. (A-D) Growth kinetics of HER2-LLC1 (A, B) and wt-LLC1 (C, D) cells in wt (A, C) or HER2 transgenic-tolerant (TG) C57BL/6 mice (B, D). A same amount (2x10^5^) of HER2-LLC1 or wt-LLC1 cells were implanted in the HER2-transgenic/tolerant or in wt-mice. Tumor growth was monitored until tumors reached an average size of 1500 mm^3^. Statistical significance was calculated using the RM (repeated measures) two way ANOVA-test (until d 27). (E) Tumor volumes at d 25 after implantation. Statistical significance was calculated using the t-test. (F) Kaplan-Meier survival curves of the four groups of mice, and statistical significance calculated by the Log-rank (Mantel-Cox) test. (G) Survival of the C57BL/6 mice treated with R-LM113 and R-115, as measured with a Kaplan-Meier survival curve. 3 wt mice and 3 TG mice were injected i.p. with 1x10^8^ or 2x10^9^ PFU of the indicated viruses. Wt or TG mice could not be differentiated one from the other and are plotted together. Statistical significance was calculated using the Log-rank (Mantel-Cox) test. (H) R-115 biodistribution to the indicated organs following four intratumoral administrations (1x10^8^ PFU/dose or vehicle), started at d 10 after tumor implantation. The indicated organs were explanted at d 26. The R-115 genome copy numbers were determined by qPCR in comparison with a standard curve obtained with purified HSV DNA, and expressed as gc/100ng of DNA. (Number of analyzed specimens. Blood, n = 15 R-115; n = 3 vehicle-treated mice. Organs, n = 4 R-115; N = 2 vehicle-treated mice).

As mentioned in the preceding paragraph, the selected mice were the C57BL/6 HER2-transgenic/tolerant mice [61]. To provide formal evidence of HER2 tolerance, we evaluated the engraftment efficiency of HER2-LLC1 or wt LLC1 cells in the HER2-transgenic/tolerant and in the wt C57BL/6 mice. Fig 2A and 2B shows that the HER2-LLC1 cells exhibited a reduced tumor growth and an about 50% reduction in the engraftment ability in wt mice relative to HER2-transgenic/tolerant mice, quantified as reduced tumor growth at d 25 (Fig 2E), and in the Kaplan-Meier survival curves (Fig 2F). In contrast, when the wt-LLC1 cells were implanted in the two types of mice, there was no substantial difference (Fig 2C–2F). Thus, the wt mice, but not the HER2-transgenic/tolerant mice, exhibited a resistance to the HER2-LLC1; the resistance was not put in place against the wt-LLC1 cells. We conclude that the HER2-transgenic mice were indeed tolerant to HER2.

The family of HER2-retargeted oHSVs exhibits a high safety profile in nude mice, by virtue of the tropism detargeting from the natural receptors, and retargeting to HER2 [42]. We asked whether the high safety profile was maintained in the immunocompetent (wt-C57BL/6) mice, and in the HER2-transgenic/tolerant counterparts. We injected wt-HSV-1(F), R-LM113 and R-115 i.p. in wt- and HER2 transgenic/tolerant mice. Since there was no difference between the HER2-transgenic/tolerant and the wt-mice, the results for the two types of mice are presented cumulatively. HSV-1(F) killed 5/6, and 2/6 mice injected with 2x10^9^ or 1x10^8^, respectively (Fig 2G). Thus, the LD_50_ was about at least 2 orders of magnitude higher than expected. A LD_50_ higher than 1x10^6^ PFU was reported for wt HSV-1 in wt-C57BL/6 upon i.p. administration [62]. Of note, none of the mice injected with R-LM113 or R-115 died, irrespective of whether they were wt or HER2 transgenic/tolerant. The results extend to immunocompetent mice the high safety profile of the HER2-retargeted oHSVs [42]. Furthermore, we evaluated R-115 biodistribution to blood and a number of organs, upon 4 consecutive intratumoral injections at 3–4 days distance. Fig 2H shows that no viral genome was detected in any of the organs.

### Higher antitumor efficacy of the mIL-12-armed R-115 relative to the non-armed R-LM113 upon early treatment schedule

**Fig 3:**
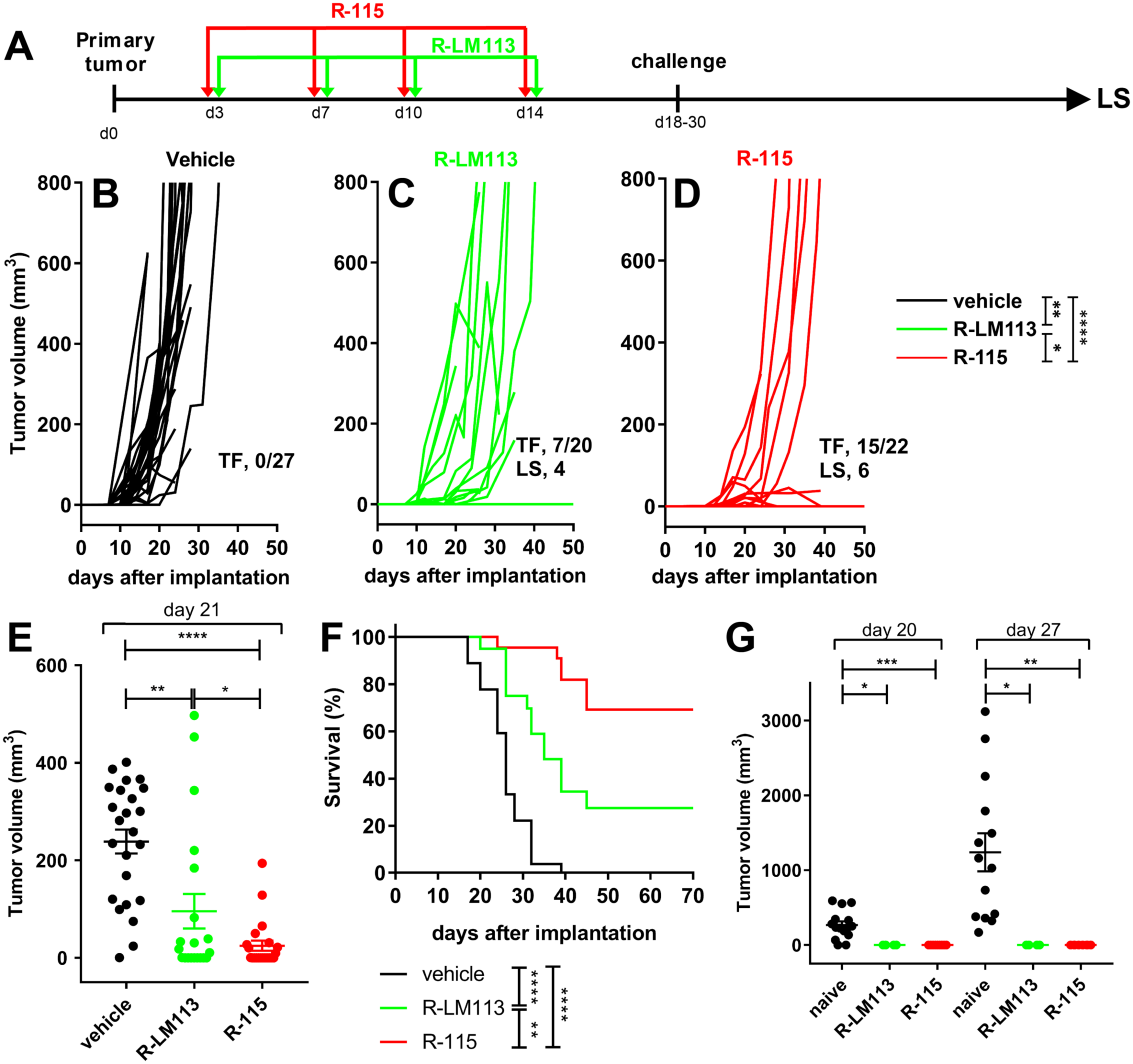
Efficacy of R-LM113 and R-115 administered early after tumor implantation on the growth of HER2-LLC1 tumors. (A) Schedule of the treatments. The HER2-transgenic/tolerant mice, implanted with HER2-LLC1 cells, received four loco-regional injections of R-LM113, R-115, or vehicle, at 3–4 days distance, starting at d 3 after tumor implantation. At d 18 or 30, mice received a 1° contralateral challenge tumor. The mice which survived the primary tumor were all resistant to the 1° contralateral challenge tumor; a fraction of them was subsequently analyzed as long survivors (LS) (see, Fig 5). (B-D) Kinetics of tumor growth in mice treated with vehicle (B), R-LM113 (C), R-115 (D). Pooled results from 3 experiments. Statistical significance was calculated using the RM (repeated measures) two way ANOVA-test (until d 21). The figures in panels B-D denote the numbers of tumor free/treated mice (TF), and the mice subsequently analyzed as LS. (E) Volumes of the primary tumors at d 21 after implantation. Statistical significance was calculated using the t-test. (F) Kaplan-Meier survival curves of the three groups of mice. Statistical significance was calculated using the Log-rank (Mantel-Cox) test. Of note, some tumor free mice were sacrificed during the course of the experiment in either arm and were censored. (G) Volumes of 1° contralateral untreated tumors in the R-LM113 and R-115 arms, and in naïve mice, at d 20 and 27 after its implantation. Statistical significance was calculated by means of the t-test. The number of mice in the naïve, R-LM113 and R-115 arms were 15, 4, 8, and 14, 4, 7 at d 20 and 27 after implantation of the contralateral tumor, respectively. The mice decreased in number because of deaths caused by the primary tumor. Four and 6 mice in the R-LM113 and R-115 arms, respectively, survived the primary tumor, received the 1° contralateral tumor and were included in the LS group (Fig 5).

The HER2-LLC1 cells were implanted in the right flanks of HER2-transgenic/tolerant C57BL/6 mice. Mice received 4 locoregional injections of R-LM113 or R-115, at three-four days distance, starting at d 3 after tumor implantation (early treatment) (see schedule in Fig 3A). The tumor growth was delayed or halted in some of the mice receiving R-LM113 relative to vehicle-treated mice (compare Fig 3C to 3B). 7/20 mice were tumor-free (Fig 3C). In the R-115-treated mice the tumor growth regressed in 8 animals, or its engraftment was inhibited, resulting in 15/22 tumor-free animals (Fig 3D). Comparison of the tumor volume at d 21 after virus treatment showed a statistically significant reduction in R-LM113-treated mice, and a higher reduction in R-115-treated mice (Fig 3E). The difference between the two treatment arms was statistically significant, and highlighted the IL-12 contribution. The Kaplan-Meier survival curves showed highly significant differences among the three treatment groups (Fig 3F). At d 18 or 30, a subset of the virus-treated mice (R-LM113, n = 4; R-115, n = 8) were implanted with a 1° contralateral challenge tumor in the opposite flank; the tumor volumes at d 20 and d 27 after its implantation is reported in Fig 3G. The key finding was that all the mice which survived the primary tumor were protected from the challenge tumor (Fig 3G), irrespective of whether they were treated with R-LM113 or R-115. We conclude that the early viral treatment induced a reduction in tumor growth. R-115 was clearly and statistically more effective than R-LM113. All mice which survived the primary tumor were resistant to a 1° contralateral challenge tumor. Some of the mice which had survived the primary tumor and had received the 1° challenge were included in the long survivor group. The relatively high amounts of viruses administered at each dose were not surprising in view of the results in Fig 1H and 1I, which indicate that it takes almost 20 PFUs (as titrated in SK-OV-3) to infect a single HER2-LLC1 cell, and that both viruses replicated in HER2-LLC1 at 1–2 log lower yields than in the human SK-OV-3 cells.

### The IL-12-armed R-115 is more effective than R-LM113 also upon a late treatment

**Fig 4:**
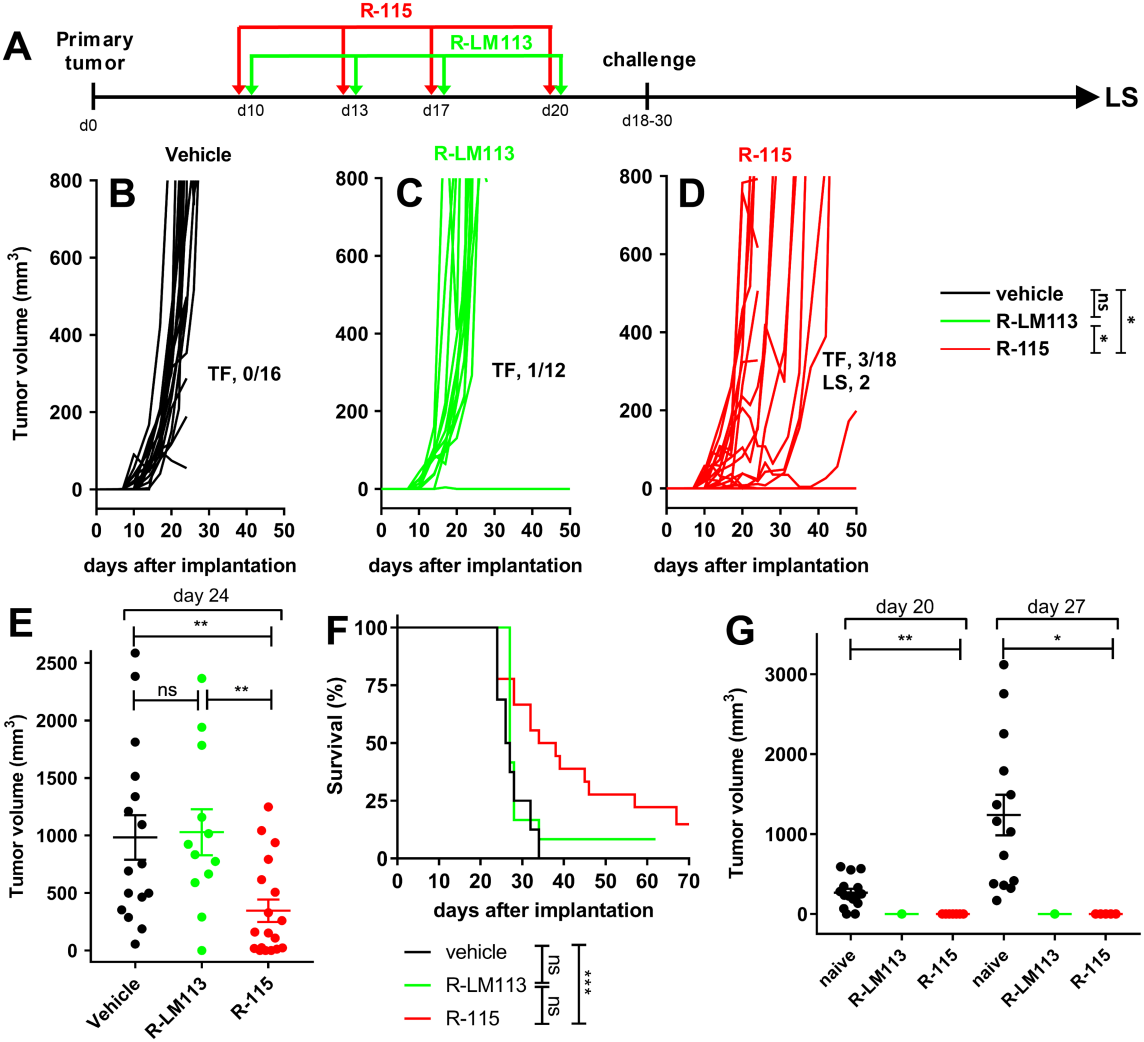
Efficacy of R-LM113 and R-115 administered 10 days after tumor implantation. (A) Schedule of the treatments. HER2-transgenic/tolerant mice implanted with HER2-LLC1 tumor cells received four peri- intra-tumoral injections of R-LM113, R-115, or vehicle, at 3–4 days distance, starting at d 10 after tumor implantation. At d 18–30 they received a 1° contralateral challenge tumor. The mice which survived the primary tumor were all resistant to the 1° contralateral challenge tumor; a fraction of them was subsequently analyzed as long survivors (LS) (see, Fig 5). (B-D) Kinetics of tumor growth in mice treated with vehicle (B), R-LM113 (C), R-115 (D). Pooled results from 2 experiments. Statistical significance was calculated using the RM (repeated measures) two way ANOVA-test (until d 24). The figures in panels B-D denote the numbers of tumor free/treated (TF) mice, and the number of mice subsequently analyzed as LS. (E) Volumes of the primary tumors at d 24 after implantation. Statistical significance was calculated using the t-test. (F) Kaplan-Meier survival curves of the three groups of mice. Statistical significance was calculated using the Log-rank (Mantel-Cox) test. Of note, some tumor free mice were sacrificed during the course of the experiment in either arm and were censored. (G) Volumes of 1° contralateral untreated tumors in the R-LM113 and R-115 arms, and in naïve mice, at d 20 and 27 after its implantation. Statistical significance was calculated by means of the t-test. The number of mice in the naïve, R-LM113 and R-115 arms were 15, 1, 7, and 14, 1, 5 at d 20 and 27 after implantation of the contralateral tumor, respectively. The mice decreased in number because of deaths caused by the primary tumor. Two mice in the R-115 arm survived the primary tumor, received the 1° contralateral tumor and were included in the LS group (Fig 5).

**Fig 5:**
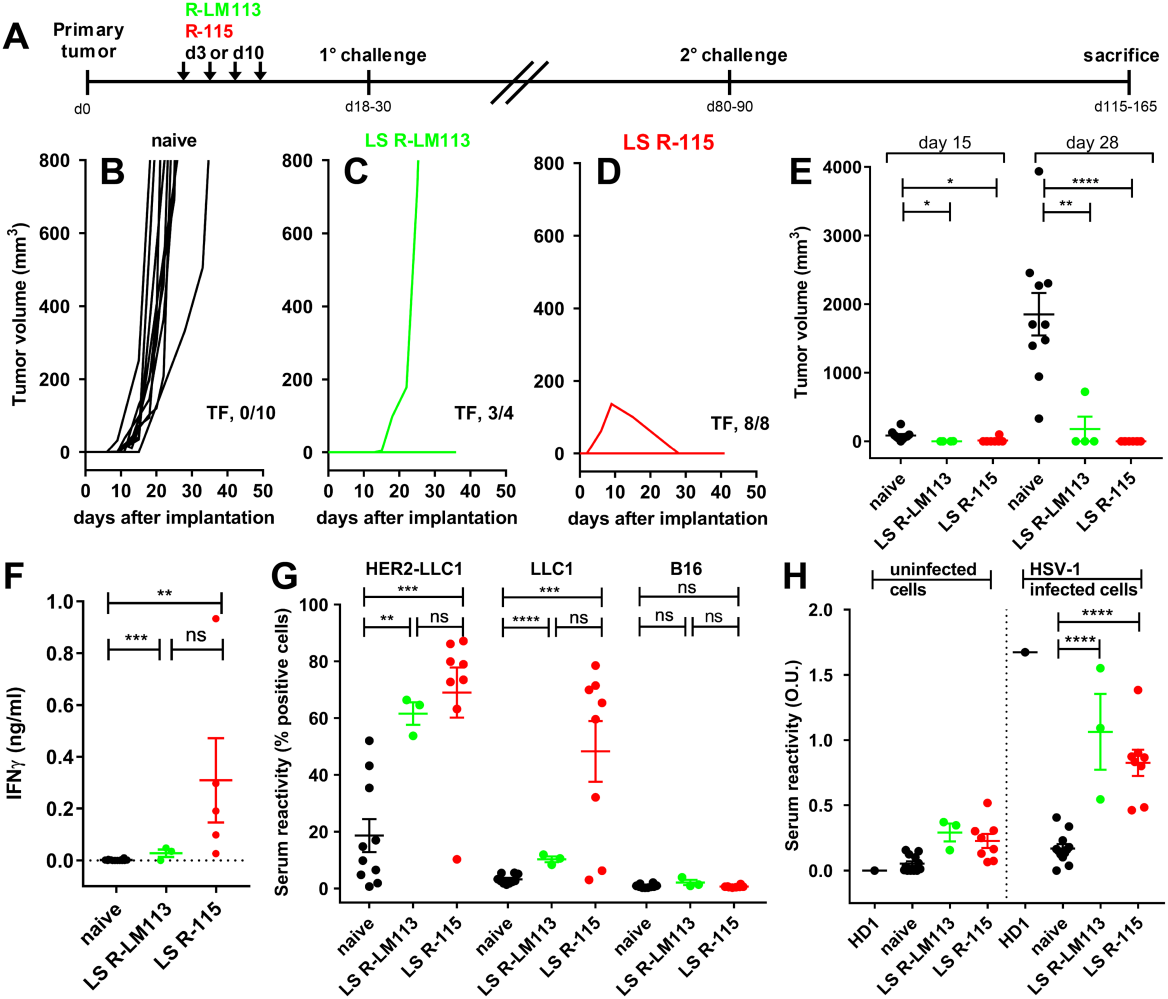
Lack of engraftment of the 2° contralateral untreated tumor in the long-term survivors from the experiments depicted in Figs 3 and 4, and systemic immune response in splenocytes and sera. (A) Overall schedule of the treatments. Long-term survivors from R-LM113 and R-115 treatments received the implantation of a 2° contralateral untreated tumor at d 80–90 after implantation of the primary tumor. (B—D) Kinetics of 2° contralateral tumor growth in the naïve (B) mice and in the long-term survivors from the R-LM113 (C) and R-115 arms (D). The figures in panels B-D indicate the number of tumor-free/treated (TF) mice. (E) Volumes of the 2° contralateral tumors at d 15 and d 28 after their implantation. (F) Immune response in splenocytes from long-term survivors. Splenocytes were incubated with HER2-LLC1 cells. Activation was quantified as IFNγ secretion in the culture medium. (G) Antibody reactivity to HER2-LLC1, LLC1 and unrelated B16 cells in the sera of the long-term survivors and of the naïve mice. The description of the assay is reported in Materials and Methods. (H). Serum Abs to HSV-1 in the long-term survivors and in the naïve mice, determined by cell enzyme-linked immunosorbent assay (CELISA), as detailed in Materials and Methods. (E—H) Statistical significance was calculated by means of the t-test.

Mice implanted with HER2-LLC1 tumor cells received 4 consecutive peri- intra-tumoral injections of R-LM113 or R-115, starting at 10 days after implantation (Fig 4A). The kinetics of tumor growth are shown in Fig 4B–4D. The R-115-treated mice exhibited a delay or reduction in tumor growth, and 3/18 were tumor-free. A significant reduction in tumor volume at d 24 is documented in Fig 4E, relative to vehicle-treated and to R-LM113-treated mice (Fig 4C–4E). In the latter group 1/12 was tumor-free (Fig 4C). The survival curves show a statistically significant difference between R-115- and R-LM113-treated mice, or control mice (Fig 4F). At d 18 or 30 mice were implanted with a 1° contralateral challenge tumor in the opposite flank, which was rejected in 100% of the animals. The volumes of the challenge tumors, and of the tumors in naïve mice is reported in Fig 4G. These experiments show that (i) R-115 treatment was more effective than R-LM113 treatment. This confirms and extends the early treatment data, and highlights a role of IL-12. (ii) All the mice which survived the primary tumor developed a durable resistance and were spared from the 1° challenge tumor. Some of the mice which had survived the primary tumor and had received the 1° challenge were included in the long survivor group analyzed in Fig 5. As mentioned, above, the moderate efficacy on primary tumor likely reflects the low susceptibility/permissivity of HER2-LLC1 cells to the viruses.

### Systemic antitumor immunity in long-term survivors from R-LM113 and R-115 therapy

The long-term survivor (LS) group included mice which survived the primary tumor, and were unaffected by the 1° challenge (see, Figs 3 and 4) (n = 4 and n = 8 in the R-LM113 and in the R-115 arms, respectively). At d 80–90 after primary tumor implantation, they received the implantation of a 2° untreated contralateral HER2-LLC1 tumor (Fig 5A for a schematic diagram of treatments). 3/4 mice in the R-LM113 arm, and 8/8 in the R-115 arm were fully protected, whereas the naïve mice were not (Fig 5B–5D). The size of the second contralateral tumor at d 15 and 28 after implantation shows no or reduced tumor growth in mice of both arms, with highest difference in the R-115-treated mice (Fig 5E).

The splenocytes from the long-term survivors which survived the 2° challenge were incubated with the HER2-LLC1 tumor cells to detect tumor-specific reactivity; 5 samples (63%) in the R-115 arm, and all 3 samples in the R-LM113 survivors’ group exhibited a significant IFNγ response (Fig 5F), and a tendency towards higher reactivity in the R-115-treated mice. In the three long-term survivors which did not show a significant response at sacrifice, but resisted tumor engraftment, the sacrifice may have taken place too long after the second challenge, or our assays were not sufficiently sensitive.

At sacrifice, the sera of the long-term survivors that resisted the 2° challenge exhibited a statistically significant reactivity to HER2-LLC1 and to wt-LLC1 cells in both treatment groups, and higher reactivity in the R-115-treated mice (Fig 5G). No reactivity was observed towards the unrelated murine tumor B16 cells, highlighting the specificity of the response (Fig 5G). The results underscore a systemic tumor-specific cell-mediated and humoral immune response in the long-term survivors, argue that the protection from distant tumor growth was immune-mediated, and suggest that the viral treatments broke the tolerance to HER2 as well as to the tumor neoantigens.

Next, we evaluated whether mice developed a long-term antibody response to HSV-1. The long survivor sera were reacted with HSV-1-infected or uninfected rabbit skin cells. As a positive control, we included a monoclonal antibody to HSV-1 glycoprotein D, named HD1 [63]. The mice treated peri- intra-tumorally with R-115 or R-LM113 developed antibodies to the virus (Fig 5H).

### R-LM113 and R-115 elicit intratumoral infiltration and activation of effector cells and their increase in spleens

**Fig 6:**
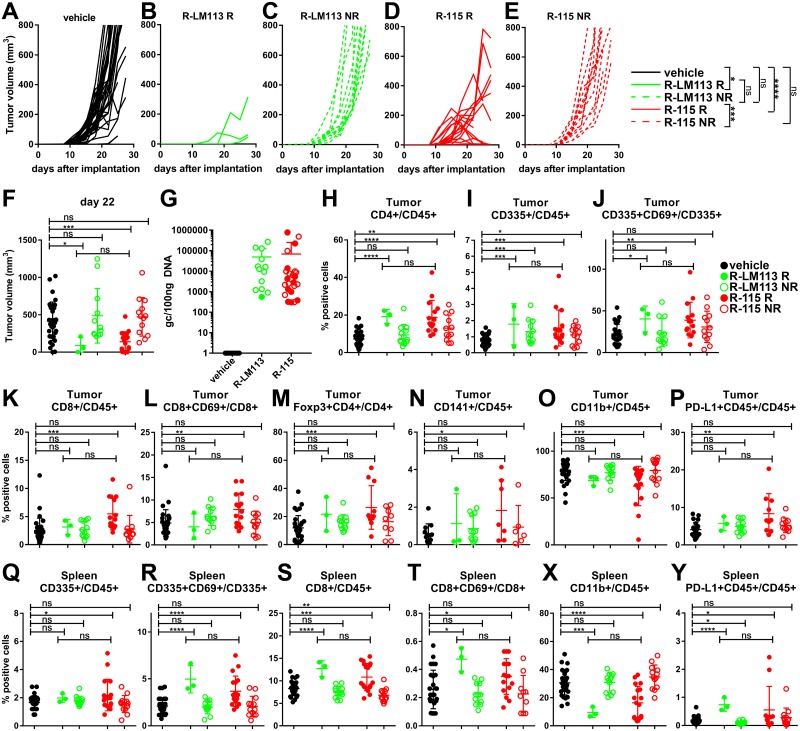
Short term intratumoral immune response and quantification of viral genome copy numbers. A new group of mice treated with R-LM113 or R-115 from d 10 after tumor implantation (same treatment schedule as in Fig 4A), was sacrificed 6–7 days after completion of the virus treatment. (A-E) Kinetics of tumor growth for mice treated with vehicle (A), R-LM113 (B, C) or R-115 (D, E). Pooled results from 3 experiments. The virus-treated mice were subdivided in responders (R) and non-responders (NR). The responders showed a regression or slowdown in tumor growth, measured as a reduction in the tumor volume in at least one of the last two measurements before sacrifice (d 2 and d 5 after the last treatment) or increments of the tumor sizes smaller than 50% in the last two measurements. The non responders exhibited a tumor growth similar to that in the vehicle-treated arm. R-LM113 responders (B) and non responders (C). R-115 responders (D) and non responders (E). Statistical significance was calculated using the RM (repeated measures) two way ANOVA-test until d 25. (F) Tumor volumes at d 22. In this and subsequent panels, Black circles, vehicle-treated mice (vehicle). Full green circles, R-LM113 responder mice (R-LM113 R). Open green circles, R-LM113 non-responder mice (R-LM113 NR). Full red circles, R-115 responder mice (R-115 R). Open red circles, R-115 non-responder mice (R-115 NR). (G) Viral genome copy number in tumors, relative to a standard curve prepared by means of purified HSV DNA. Results are expressed as gc/100ng of DNA. (H-O) Tumor infiltrating cells. (Q-X) Splenocytes. (P, Y) PD-L1 expression by CD45+ cell in tumors (P) and in spleens (Y). (F, H-Y) Statistical significance was calculated using the t-test.

To shed light on the immune basis of the acquired resistance and to identify the immune effectors associated with the therapeutic effects exerted by R-LM113 and R-115, we evaluated the modifications elicited by the viruses in tumor infiltrating cells and in immune markers. A new group of mice was treated as depicted in Fig 4A, i.e. with 4 consecutive virus administrations, starting at d 10 after tumor implantation. Tumors were explanted 6–7 days after completion of the virus treatment. Based on the virus effect on tumor growth, we subdivided the animals in responders and non-responders. The first exhibited a regression or slowdown in tumor growth. The latter exhibited a tumor growth similar to that in the untreated arm. The tumor growth curves are shown in Fig 6A–6E. A most striking result was the difference in the responder/non-responder ratio between the two arms, namely 3/12 and 18/13 in the R-LM113 and R-115 arms. This corresponds to 20% and 58% responders/treated animals in the two arms. The tumor volumes in the five groups at d 22 is reported in Fig 6F. HSV genome copies in the tumors ranged from 300 to 775000 copies/100ng DNA, did not differ between the two treatment arms, nor between responders and non-responders, and was in overall agreement with previous observations [36]. In the blood and in a number of tissues, there was practically no detectable virus genome, as shown in Fig 2H. Thus, virus presence was strictly limited to the tumor. The presence of non-responder mice may reflect both the stochastic nature of the immune response and individual variations in the repertoire of immune cells, and the fact that the amounts of administered viruses were insufficient to elicit a total response, as seen in the efficacy data.

Next, we characterized the immune cells present in tumors and spleens. Although the number of responder mice in the R-LM113 arm was very small, and data should therefore be interpreted cautiously, two patterns emerged. Thus, CD4+ and CD335+ cells—both total and expressing the CD69 activation marker—were increased in the tumors of all responder mice (Fig 6H–6J). CD8+, both total and expressing the CD69 activation marker, Foxp3+, and CD141+ cells were increased and CD11b+ cells were decreased preferentially in the responder R-115-treated tumors (Fig 6K–6O). The distribution of these cells in the spleens mirrored that in the tumors (Fig 6Q–6X). PD-L1 was increased in tumor from R-115-treated mice, and in the spleens of mice treated with either virus (Fig 6P and 6Y). In as much as PD-L1 expression is regulated downstream of the IFN R signaling, its increase in the responder group was likely induced at least in part by IFNγ. The results suggest that some changes to tumor infiltrating and spleen infiltrating lymphocytes/monocytes were likely elicited by either virus, and, conversely, that the CD8+ cell infiltration was preferentially induced by the IL-12 encoding R-115.

### Intratumoral cytokine profile and Th1 signature

**Fig 7:**
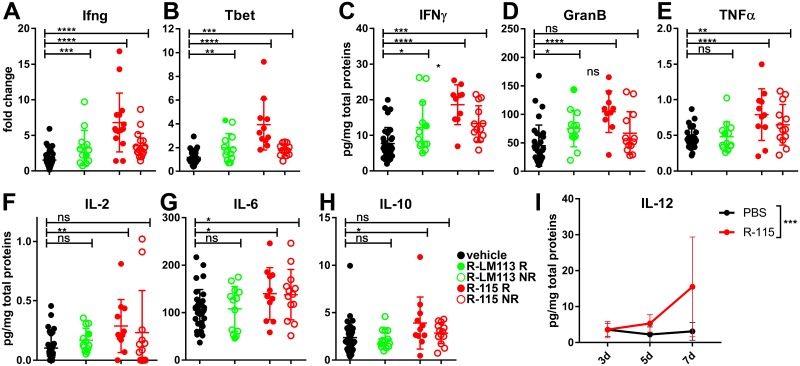
Transcriptional analysis of tumor specimens and quantification of intratumoral cytokines and molecules. (A-B) qRT-PCR determination of expression of Ifng and Tbet genes in tumor specimens from mice described in Fig 6. Statistical significance was calculated using the t-test. (C-H) Quantification of intratumoral cytokines by Luminex Multiplex Assay in mice described in Fig 6. Small tumor specimens were resuspended in non-denaturing lysis buffer and lysed by sonication. Supernatants were assayed by Luminex Multiplex Assay and results were expressed as pg of the analyte/total proteins. Statistical significance was calculated using the t-test. (I) Quantification of intratumoral mIL-12 secretion. R-115- or vehicle-treated mice were sacrificed at d 3, 5 or 7 after completion of the virus treatment (d 21). Tumors were lysed as detailed above, and mIL-12 was quantified by ELISA. Each point represents the average of 4 determinations. Statistical significance was calculated using the two way ANOVA-test.

Next, we analyzed the tumors from Fig 6 by reverse transcription quantitative PCR (qRT-PCR). The specimens from both the R-LM113- and R-115-treated mice exhibited a highly significant difference in Ifng and Tbet mRNA levels, relative to the vehicle-treated mice (Fig 7A and 7B). Tbet is a transcription factor which contributes to drive a type 1 T helper (Th1) response and controls IFNγ expression. There was a clear tendency towards higher response in the R-115 arm, even though a statistical significance analysis was not carried out, given that a single sample was present in the responder R-LM113 group. The results provide a second line of evidence for activation of the intratumoral immune response elicited by the retargeted oHSVs, with a trend for higher activation in the R-115 responder mice.

An unbiased analysis of the immune markers at the protein level was carried out by Luminex Multiplex, in tumor specimens taken at 6–7 days after virus treatment. Even in this assay, the number of available specimens for the responder R-LM113-treated mice was too low; therefore the R-LM113-derived specimens were considered as a single group. In specimens from both virus treatments there was a clear increase in IFNγ and Granzyme B, especially in the R-115 responders (Fig 7C and 7D). There was an increase also in TNFα and IL-2 in the R-115 specimens (Fig 7E and 7F), highlighting a polarization towards a Th1 response and an activation of the effector cells. IL-10 and IL-6 were specifically increased in the R-115 specimens (Fig 7G and 7H). The IL-12 was detectable at d 5 and increased at d 7 after completion of R-115 treatment (Fig 7I). In R-LM113-treated mice no increase in IL-12 was observed at d 5 relative to vehicle-treated mice. The data extend the evidence for a de-repression of the immunosuppressive microenvironment induced especially by R-115. We note that TNFα, IL-6 and IL-10 are induced also by HSV, so their presence may in part be consequent to infection [54,55,57].

## Discussion

We report on the first efficacy studies of fully virulent, HER2-retargeted oHSVs, either unarmed (R-LM113) or armed with mIL-12 (R-115). Their peri- intra-tumoral administration led to a reduction in the growth of the primary tumor, particularly in the R-115-treated mice, to intratumoral and systemic immune responses which almost completely prevented the engraftment of distant untreated tumors. In essence, the intralesional vaccination promoted local and systemic immunity. Pertinent to the model system and the main results is the following.

Inasmuch R-LM113 and R-115 were retargeted to HER2, the murine cancer cells were made HER2-transgenic, and the mice were the HER2-transgenic/tolerant C57BL/6 [61]. The safety profile of both viruses was very high, as mice resisted i.p. doses as high as to 2x10^9^ PFU, which were lethal for 83% of the mice injected with wt-HSV. Furthermore, upon peri- intra-tumoral delivery, there was no detectable R-115 infection in organs other than the injected tumors. The selected tumor cells were the HER2-LLC1, which are markedly less sensitive/permissive than the human cancer cells. This animal system is therefore adequate to investigate the immunotherapeutic efficacy of the HER2-retargeted oHSVs, but is hardly predictive of the therapeutic effects on primary tumors. The high resistance of murine tumor cells to HSV is shared with a large part of preclinical studies on the therapeutic effects of oHSVs [58,59], and clearly results in underestimation of the oncolytic and immunotherapeutic effects of oHSVs.

The key efficacy and immunotherapeutic data were as follows. The IL-12 armed R-115 reduced and delayed tumor growth more efficiently than the unarmed R-LM113. Practically all the mice which survived the primary tumor—from either the R-LM113 or R-115 arm—were protected from a distant challenge tumor, and, after several weeks, from a subsequent re-challenge. Hence, the highest effect was the abscopal one. A specific systemic immune response was detected at sacrifice both in splenocytes and in sera of the long-term survivors. The durable vaccine effect adds to the strong oncolytic effects exerted on the primary human tumors in immunodeficient mice [42,50,51].

The immune basis of the therapy was documented by two lines of evidence, i.e. the early modifications to the immune suppressive tumor microenvironment and the establishment of a systemic response. With respect to the early modifications, the tumors in responder mice contained significant amounts of immunostimulatory factors, at higher frequencies/amounts in the R-115 arm. Beside IL-12, these included IFNγ, IL-2, Granzyme B, Tbet and TNFα,—typical effectors of IL-12, Th1 polarization and natural killer (NK) cell activation, in addition to IL-10 and IL-6. The tumor microenvironment of the responder mice was further characterized by infiltration and activation of immune cells. Although the number of R-LM113 responders was very low and data should be considered cautiously, two patterns emerged. CD4+ and CD335+ NK cells appeared to be induced by either virus. These cells may well represent the first line of anti-tumor defence put in place by virulent oHSVs. CD8+ and CD141+ cells, PD-L1+ tumor cells, and Foxp3 T regulatory cells were preferentially induced by R-115, along with a decrease in the number of intratumoral CD11b+ cells. The role of each immune cell subpopulation of in virus-induced antitumor activity is the subject of intense studies. The impact of the CD8+ cell infiltration in unleashing the immunosuppressive phenotype of the tumor and in sensitizing otherwise refractory cancers to checkpoint inhibitors was documented recently also in clinical specimens from patients treated with Oncovex^GM-CSF^ + pembrolizumab [18,64]. More controversial appears to be the role of NK cells. These cells and their effectors IFNγ, TNFα, Granzyme B were selectively increased in the R-115-treated tumors. The impact of the NK cell recruitment in the Maraba virus-induced eradication of cancer was well established [34]. In contrast, in glioblastoma viro-immunotherapy induced by a transcriptionally retargeted oHSV the activation and recruitment of NK cells to the treated tumor diminished the anti-tumor efficacy [65]. Specific NK cell depletion experiments will shed light on the role played by NK cells on the antitumor activity of the HER2-retargeted o-HSVs. The increase in Foxp3+ T-regulatory lymphocytes was observed upon treatment with other OVs, e.g. Newcastle disease virus, and may reflect an autoregulatory mechanism put in place upon CD8+ cell-mediated response [66]. Also the monocytic lineage was affected by the viral treatment; in particular CD11b+ cells were decreased in R-115 responders. These cells include cells that contribute to the immunosuppressive properties of the tumor microenvironment, including myeloid-derived-suppressor cells [67]. Not surprisingly they were present in higher amounts in the tumors of non responder mice, and may contribute to resistance. Of the cytokines induced by R-115, TNFα, IL-6 and IL-10 are part also of the signature of the antiviral innate response to HSV, finalized to limit the virus-hostile microenvironment and to favour virus replication [53–55,57]. Hence, the immune-related molecules in the tumor microenvironment are likely to be induced in part by the virus itself, in part by the virus-encoded IL-12. The former are not necessarily detrimental to the control of tumor growth. It has been proposed that the OV-induced antiviral immune responses may exert intrinsic anticancer benefits and may be critical for establishing clinically desired antitumor immunity [68,69]. Studies with a recombinant o-poliovirus suggested activation of dendritic cells and immune adjuvancy as a result of canonical innate anti-pathogen response [70].

The systemic response was detected in splenocytes and as durable antibody response towards both the HER2-LLC1 and the wt-LLC1 cells. This finding implied that the virus treatment broke the tolerance not only to the HER2-LLC1 but also to the wt-LLC1 cells, and argued that, especially for R-115, a systemic route of delivery was not required to achieve a systemic vaccine protection. The high efficacy against the engraftment of distant untreated tumors, implanted long after virus treatment, and the Th1 polarization bare strong similarities with the therapeutic effect of a IL-12-armed oncolytic measles virus [33]. In that case, the viral backbone was that of a measles virus vaccine strain.

Differences are readily evident between current efficacy data and those exerted by attenuated Δγ_1_34.5 oHSVs. The major effect of Oncovex^GM-CSF^ on B16 melanoma cancer cells in C57BL/6 mice was exerted on the primary treated tumor. The effects on the distant untreated tumors were limited [58] and a long-term protection was only achieved when the Oncovex^GM-CSF^ was combined with anti-CTLA-4 [58]. The different effects seen in that study and in current investigation likely reflect the different action of the cytokines expressed by the respective oHSV, i.e. the GM-GSF in Oncovex^GM-CSF^ and the IL-12 in R-115, the permissivity of the respective murine cancer cells to HSV infection, as well as the genomic properties of the viral backbones, namely a deleted/attenuated viral genome in Oncovex^GM-CSF^, and a full non-attenuated genome in R-115. The importance of the genomic backbone is clearly apparent when the effects elicited by R-115 are compared to those elicited by the IL-12-armed G47 [19]. The two recombinants express the same cytokine, and differ strikingly in their genome. The IL-12-G47 carries multiple deletions, whereas R-115 carries no deletion. As a monotherapy against a model glioblastoma tumor, G47 alone or the IL-12-G47 were barely effective [19]. A strong efficacy required the combination of IL-12-G47 with both CPIs, the anti-CTLA-4 and anti-PD-1 Abs. While the glioblastoma model likely exemplifies a refractory and immunosuppressive tumor, and, possibly, a resistant tumor to HSV infection, the current finding that the IL-12-armed R-115 reduced the growth of the primary tumor and fully protected the mice from the growth of distal tumors in the absence of combination with CPIs argue that the fully virulent retargeted oHSVs have the potential to be highly active oncolytic-immunotherapeutic agents.

## Materials and methods

### Cells and viruses

Human ovary SK-OV-3 cancer cells (Roswell Park Memorial Institute) were cultured in RPMI- Glutamax (Life Technologies #61870–010) containing 10% fetal bovine serum (FBS). LLC1 and B16 cells were purchased from ATCC and cultured in Dulbecco modified Eagle medium (Life Technologies, #31966–021) containing Glutamax, High Glucose and 10% FBS.

R-LM113 was described [44]. R-115 is a derivative of R-LM113; like the parental R-LM113, it is retargeted to HER2. It expresses the murine IL-12 (mIL-12) under the CMV promoter [60]. Viruses were cultivated and titrated by plaque assay in SK-OV-3 cells. The mIL-12 production was quantified in the supernatant of R-115-infected cells by means of mIL-12 ELISA kit (EMIL12, Thermo Fisher Scientific) according to manufacturer instruction.

### HER2-transgenic murine cancer cells

The HER2 receptor expressed in murine cancer cells was a chimera in which the C-tail signaling portion was replaced with the C-tail of the non-signaling nectin1 receptor. The construct was named as HER2-nectin, and herein referred to as HER2. For the engineering, the ectodomain of human HER2 receptor was amplified from pcDNA-HER2 plasmid [71] with primers HER2_NheI_f GCGGCCGCGCTAGCATGGAGCTGGCGGCCTTGTGCCGC and HER2_HpaI_r AGAGATGATGGAGTTAACAGGGCTGGCTCTCTGCTCGGCGGG. The HER2 ectodomain amplicon spans from ATG start codon up to the amino acid 650 (GenBank NM_004448). pCF18HNK was described [72]. The plasmid was cut with NheI-HF and HpaI (New England Laboratories) to delete the ectodomain of human nectin1 from amino acid 1 to 330 (GenBank NM_203285.1 aa 1–330). The HER2 ectodomain amplicon was cut with NheI and HpaI and then ligated into NheI/HpaI digested pCF18HNK to generate pHER2-Nectin plasmid. The entire HER2-Nectin chimeric ORF was sequenced. Then, a NheI/XbaI fragment from pHER2-nectin plasmid was subcloned in the NheI/XbaI digested lentiviral expression vector pLV-EF1-MCS-SV40-Puro. The resulting expression vector was called pLV-HER2-nectin-puro.

The B16 melanoma and the LLC1 cells were made transgenic for HER2-nectin expression by lentiviral transduction, as detailed [73]. Transduced cells were selected by means of puromycin, enriched for HER2 expressing cells with microbeads (Miltenyi Biotech) following incubation with an anti-HER2 mouse IgG antibody (SantaCruz Biotecnology, clone 9G6). Single-cell clones were obtained by limiting dilution. Clones were checked for stable HER2 expression for up to 30 passages in cell culture by flow cytometry (BD Accuri) with anti-HER2 MGR2 antibody (Vinci-Biochem, #ALX-804-573-C100).

### Virus growth and plating efficiency

To measure the virus growth, HER2-LLC1, HER2-B16 and SK-OV-3 cells were infected at an input multiplicity of 0.1 PFU/cell (as titrated in the respective cell line) for 90 min at 37°C. Unabsorbed virus was inactivated by means of acidic wash (40 mM citric acid, 10 mM KCl, 135 mM NaCl, pH 3). Replicate cultures were frozen at the indicated times (24 and 48 h) after infection and the progeny was titrated in SK-OV-3.

To determine the relative plating efficiency, replicate aliquots of R-115 or R-LM113 were plated onto HER2-LLC1, HER2-B16 and SK-OV-3 cells. The infected monolayers were overlaid with medium containing agar. The plaques were scored 3 days later. The results represent the average of triplicates ± SD.

### In vivo experiments

C57BL/6 mice transgenic for and tolerant to HER2 (B6.Cg-Pds5bTg(Wap-ERBB2)229Wzw/J) [61] were obtained from Wayne State University through The Jackson Laboratories, and bred in the facility of the Department of Veterinary Medical Sciences, University of Bologna. The animals for tumor implantation were HER2-transgenic (HER2-TG). HER2-LLC1 cells were implanted subcutaneously in the left flank of six-to-eight weeks old HER2-TG C57BL/6 mice in 250 μL of PBS, 0.2x10^6^ cells/mouse. The start of the virus treatment is detailed in the Results section. Mice received 4 loco-regional or peri- intra-tumoral (p.i.t.) injections of the respective virus suspension, diluted in PBS, 1x10^8^ PFU/mouse, at 3–4 days distance. Mice in the control group received PBS (vehicle). Each treatment group consisted of 5, 10 or more mice, as detailed in the Figure legend. Tumor volumes were scored twice weekly by measuring the largest and the smallest diameter by means of a calliper. Tumor volume was calculated using the formula: largest diameter x (smallest diameter)^2^ x 0.5. Mice were killed when tumor volumes exceed 1000–2000 mm^3^, ulceration occurred, or animals exhibited distress or pain. Where indicated, mice received a contralateral tumor, made of HER2-LLC1 cells implanted subcutaneously in the right flank, 0.2x10^6^ cells/mouse. The contralateral tumors were not treated.

### Tumor-specific IFNγ memory response by murine splenocytes

Freshly explanted spleens were smashed through a 70 μm cell strainer in PBS with a sterile 5ml syringe plunger to isolate splenocytes. Red blood cells in spleen and tumor samples were lysed with ACK buffer (NH_4_Cl 150 mM, NaHCO_3_ 10 mM, 1mM EDTA), resuspended in medium (RPMI 1640 containing 10% heat inactivated FBS, 1% penicillin/streptomycin, 0.05 mM β-Mercaptoethanol), counted and seeded in 24 well plate. Splenocytes (1x10^6^ cell/well) were incubated with 1x10^5^ HER2-LLC1 cells in 0.5 ml medium, and cocultured for 48 h. Media were collected and the amount of secreted IFNγ was quantified by ELISA (IFN-gamma Mouse ELISA Kit, Thermo Fisher).

### Intratumoral cytokine profiling

Tumors were minced, resuspended in lysis buffer (Tris-HCl pH 7.4 50 mM, NaCl 250 mM, EDTA 5 mM, Na_3_VO_4_ 1 mM, NaF 50 mM, NaN_3_ 0.02%, Sodium deoxycholate 0.5%, NP40 1%, N^α^-p-tosyl-L-lysin chloromethyl ketone hydrochloride 0.3 mM, N^α^-p-tosyl-L-phenylalanine chloromethyl ketone 0.3 mM, PMFS 1 mM), in a proportion of 500μL of lysis buffer for 100 mg of tumor. Samples were sonicated with Bioruptor (Diagenode), using program HIGH for 20 minutes (30sec ON 30sec OFF) and centrifuged for 30 minutes at 11000 x g. The protein content of supernatants was determined by means of the Bio-Rad protein assay (Bio-Rad); the supernatants were analyzed by means of a Magnetic Luminex Assay (R&D) and a mouse premixed Multi-Analyte kit. The custom-made kit included: TNFα (BR14), IL-12 p70 (BR15), IL-2 (BR22), IL-4 (BR25), IL-6 (BR27), IL-10 (BR28), IL-17A (BR30), IFNγ (BR33), CXCL10/IP-10 (BR37), Granzyme B (BR63). Supernatants were 1:1 or 1:5 diluted with the Calibrator Diluent RD6-52 and analyzed according to manufacturer instructions. Standard curve with 1:1 diluted lysis buffer was employed to quantify TNFα, IL-2, IL-4, IL-6, IL-10, IL-17A, IP-10 and Granzyme B in 1:1 diluted samples, while the quantification of IFNγ was performed in 1:5 diluted samples, using the corresponding standard curve in 1:5 diluted lysis buffer. Data were analyzed according to the manufacturer instructions. Results were expressed as pg of each analyte per mg of total proteins. Quantification of IL-12 p70 was under the LOD for Luminex analysis, hence the analyte was quantified by means of the Mouse IL-12 ELISA kit.

### Intratumoral transcriptional response

Tumors were homogenized. A few mgs of the homogenates were employed for total RNA purification with the Nucleospin RNA kit (Macherey-Nagel) according to the manufacturer’s protocol (including the on-column DNaseI treatment). 1.2 μg of RNA was employed for the cDNA synthesis using the High-Capacity cDNA Reverse Transcription Kit (Applied Biosystems), following the manufacturer’s instruction. qRT-PCR reactions were prepared as follows: 2 μL of the diluted (1:4) cDNA samples were mixed with 5 μL of TaqMan Fast Advanced Master Mix (Applied Biosystems) and 0.5 μL TaqMan probes in a final volume of 10 μL. The TaqMan probes employed for the assay were: mm01168134_m1 (Ifng), mm00450960_m1 (Tbx21/Tbet), mm01612987_g1 (Rpl13a). qRT-PCR reactions were performed in a StepOnePlus System (Applied Biosystems), following the protocol indicated in the Master Mix. The levels of expression were determined using the ΔΔCt method, normalized on the Rpl13a housekeeping gene.

### Biodistribution of HSV genome

Tumors and organs were homogenized; a few mgs of the homogenates or 50 μL of blood were employed for DNA purification with the Nucleospin Tissue kit (Macherey-Nagel) according to the manufacturer’s protocol. HSV genome copies (gc) were quantified by qRT-PCR. 2 μL of the dilutions or 30 ng of the tissue DNAs were mixed with 5 μL of TaqMan Fast Advanced Master Mix (Applied Biosystems) and 0.5 μL HSV probe in a final volume of 10 μL. The HSV probe contained the following oligonucleotides: DnapolFw (CATCACCGACCCGGAGAGGGAC), DnapolRev (GGGCCAGGCGCTTGTTGGTGTA) and DNA_Pol_PROBE (FAM-CCGCCGAACTGAGCAGACACCCGCGC-TAMRA), 2.50μM each. qRT-PCR reactions were performed in a StepOnePlus System (Applied Biosystems), following the protocol indicated in the Master Mix. The amount of gc was determined by comparison with a standard curve, prepared using purified genomic HSV DNA, and expressed as gc/100ng of DNA.

### Serum reactivity

LLC1 and HER2-LLC1 cells were trypsinized, rinsed and resuspended in flow cytometry buffer (PBS + 2% FBS). For each sample, 0.25x10^6^ cells were reacted with mouse serum, diluted 1:60, in 96 well plate in ice for 1 hour, rinsed with flow cytometry buffer and finally incubated with anti mouse PE (1:400, Beckton Dickinson). Data were acquired on BD C6 Accuri. Cell enzyme-linked immunosorbent assay (CELISA) was performed as described [74]. Briefly, RS cells were infected with HSV-1 (F) at 3 PFU/cell, in 96 well plate. Twenty-four h later they were fixed with paraformaldehyde, reacted with mouse serum diluted 1:60, or with MAb HD1 diluted 1:400, followed by anti-mouse peroxidase. Finally, peroxidase substrate o-phenylenediamine dihydrochloride (OPD; Sigma-Aldrich) was added and plates were read at 490 nm with GloMax Discover System (Promega Corporation).

### Intratumoral and spleen infiltrating lymphocytes

Single cell suspensions were prepared from freshly isolated HER2-LLC1 tumors and spleens at sacrifice. Tumors were minced in small pieces and digested with collagenase (1 mg/ml) for 1.5 h at 37°C. The resulting cell suspensions were passed through 70 μm cell strainer and rinsed with flow cytometry buffer. Spleens were processed as described above, and then treated as the tumor samples. Red blood cells in spleen and tumor specimens were lysed by means of ACK buffer, samples were pelleted and resuspended in flow cytometry buffer. Subsequently for each sample 2x10^6^ cells were blocked with α-CD16/32 Ab (clone 93, eBioscience), and then reacted with the antibodies CD4-FITC (clone GK1.5, eBioscience), CD8a-PE (clone 53–6.7, eBioscience), CD45-FITC (clone 30-F11, eBioscience), CD45-Percp-Cy7 (clone 30-F11, eBioscience), CD335-PE (clone 29A1.4, eBioscience), FoxP3-PE (clone 150d/e4, eBioscience), CD11b-FITC (clone M1/70, eBioscience), PD-L1-APC (clone MIH5, BD), CD141-PE (clone LS17-9, eBioscience) and CD69-PercP (clone H1-2F3, eBioscience). Data were acquired on BD C6 Accuri. Only samples which provided at least 100000 events were included in subsequent analysis.

### Ethical statement

Animal experiments were performed according to European directive 2010/63/UE, Italian laws 116/92 and 26/2014. The experimental protocols were reviewed and approved by the University of Bologna Animal Care and Use Committee (‘‘Comitato per il Benessere degli Animali”, COBA), and approved by the Italian Ministry of Health, Authorization # 86/2017-PR to Prof. Anna Zaghini.

### Statistical analyses

Statistical analyses are reported in figure legends, as it applies: *, p < 0.05; **, p < 0.01; ***, p < 0.001; ****, p < 0.0001; ns, non-significant.
